# Prevalence, perceptions and predictors of alcohol consumption and abstinence among South Australian school students: a cross-sectional analysis

**DOI:** 10.1186/s12889-017-4475-5

**Published:** 2017-06-07

**Authors:** Jacqueline A. Bowden, Paul Delfabbro, Robin Room, Caroline L. Miller, Carlene Wilson

**Affiliations:** 1 Population Health Research Group, South Australian Health and Medical Research Institute, Adelaide, South Australia 5001 Australia; 2 Psychology Department, University of Adelaide, South Australia, 5005 Australia; 3 Centre for Alcohol Policy Research, La Trobe University, Melbourne, Vic 3000 Australia; 4 Centre for Social Research on Alcohol and Drugs, Stockholm University, SE, 106 91 Stockholm, Sweden; 5 School of Medicine, Flinders Centre for Innovation in Cancer, Flinders University, Adelaide, South Australia 5042 Australia; 6 Olivia Newton John Cancer Wellness and Research Centre and School of Psychology and Public Health, La Trobe University, Melbourne, 3083 Australia; 7 School of Population Health, University of Adelaide, South Australia, 5000 Australia

**Keywords:** Alcohol consumption, School students, Cancer

## Abstract

**Background:**

Alcohol consumption by young people (particularly early initiation) is a predictor for poorer health in later life. In addition, evidence now clearly shows a causal link between alcohol and cancer. This study investigated prevalence, predictors of alcohol consumption among adolescents including perceptions of the link between alcohol and cancer, and the role of parents and peers.

**Methods:**

A sample of Australian school students aged 12–17 years participated in a survey (*n* = 2885). Logistic regression analysis was undertaken to determine predictors.

**Results:**

Alcohol use increased with age and by 16, most had tried alcohol with 33.1% of students aged 12–17 reporting that they drank at least occasionally (95% CI = 31.0–35.2). Awareness of the link between alcohol and cancer was low (28.5%). Smoking status and friends’ approval were predictive of drinking, whereas parental disapproval was protective. Those aged 14–17 who did not think the link between alcohol and cancer was important were more likely to drink, as were those living in areas of least disadvantage. The only factors that predicted recent drinking were smoking and the perception that alcohol was easy to purchase.

**Conclusions:**

An education campaign highlighting the link between alcohol and cancer may have positive flow-on effects for young people, and schools should incorporate this messaging into any alcohol education programs. Consideration should be given to factors that serve to regulate under-aged accessibility of alcohol.

## Background

Alcohol consumption is responsible for approximately 3.3 million deaths annually, and accounts for 5.1% of the global burden of disease. Harmful consumption of alcohol has been ranked among the top five risk factors for non-communicable disease, disability and death globally and has been causally linked to over 200 health conditions including cancer [1]. In 1988, alcoholic beverages were classified as a class 1 carcinogen [2], and a large body of evidence now demonstrates the causal link between alcohol consumption and cancer [3]. Alcohol consumption is therefore a topic of considerable public health concern internationally.

A recent Australian study estimated that over 5500 Australian deaths are attributable to alcohol each year [4]. Among males, injury was held responsible for 36% of these deaths, followed by cancers (25%) and digestive diseases (16%). Among females the highest proportion of alcohol-attributable deaths was from cardiovascular diseases (34%), followed by cancers (31%) and injuries (12%) [4]. There is now convincing evidence that alcohol causes cancer of the mouth, pharynx, larynx, oesophagus, bowel (in men) and breast cancers among women. There is also probable evidence that alcohol increases the risk of bowel cancer in women and liver cancer [3]. There is a dose-response relationship between alcohol and cancer risk with increasing consumption associated with increased risk [3, 5]. For this reason, Cancer Councils are now recommending that ‘to reduce their risk of cancer, people limit their consumption of alcohol, or better still avoid alcohol altogether’ [6]. It is possible that these estimates may be updated and increased over time with more emerging evidence of the link between alcohol and cancer.

Drinking patterns tend to be laid down in adolescence and early adulthood [7]. Consumption by young people, particularly early initiation (i.e. for those aged 11 to 14 years), is a predictor of poorer health in later life [8]. There has been a decline in alcohol consumption among adolescents in Australia, particularly in the last decade [9], Europe and the US [10, 11]. Despite this, alcohol remains one of the most commonly used intoxicating substances among school students. It is important to note that while the number of current drinkers has decreased, the rate of consuming more than four drinks on one occasion in the past 7 days has not decreased among current drinkers [9]. Research in 2011 suggested that 50.7% of Australian secondary school students had consumed alcohol in the past year. Rates of drinking regularly increase with age from 5.1% at age 12 to 36.7% by age 17 [12].

Improving community understanding of lifestyle risk factors associated with cancer has been identified as a key strategy for preventing cancer globally [13]. Cancer is one of the most feared diseases in Australian adults [14], and internationally [15]. Improving awareness of the link between alcohol and cancer therefore may well influence an individual to consider moderation of their consumption or even abstinence. However, international evidence shows that the majority of people are not aware of the link between alcohol and cancer [16–18]. This is the case in Australia, with a recent study finding that only 36.6% of adults were aware of the important link. This study also found that those that were aware of the risk were less likely to drink beyond the health guidelines threshold for lifetime risk [19]. Only a few studies have examined awareness among young people, with a study in the UK finding that 37% of young people aged 15 to 24 years were aware of the link [18]. To our knowledge, awareness of the link between alcohol and cancer has not previously been examined among secondary school students in Australia.

Understanding adolescents’ reasons for drinking is critical for developing intervention strategies. The Social Development Model postulates influence from social controls, social learning and patterns of association (whereby attitudes and anti-social behaviours are acquired through interaction with others) as important predictors of poor and good behavioural choices in adolescence [20]. Consistent with this model, alcohol consumption in young people has been associated with parental attitudes toward consumption [21], peer use, and perceptions of peer attitudes to alcohol use [22]. According to this model, the influence of peers becomes increasingly important in later adolescence, at which time parental involvement and the influence of family declines [20]. The role of peer influence, particularly in later adolescence has been supported in both theory and empirical alcohol studies [23].

Alcohol consumption among school children has been associated with a number of other covariates, including: more weekly spending money [24]; self-reported academic difficulty among females [25]; and participation in other risk-taking behaviours including smoking [26]. The relationship between alcohol consumption and socio-economic status (SES) is less clear than it is with other risk factors for cancer. People with higher SES tend to drink more frequently than others, but among those that drink, the lower socio-economic groups tend to drink larger quantities [27].

The first aim of this study is to confirm currently documented prevalence of alcohol consumption and it is hypothesised, that consistent with most recent evidence, there will be a pattern of increasing consumption with age among adolescents. The principle hypothesis is that the majority of students will not be aware that alcohol causes cancer but among those that are, will be less likely to drink alcohol or be recent drinkers. The second hypothesis of this study is that perceived parental disapproval of alcohol consumption will be a protective factor for consumption in later school years.

## Methods

### Study population

Data were obtained from a 2011 cross-sectional survey of a representative sample of South Australian secondary students that formed part of a larger cohort, namely the Australian School Students’ Alcohol and Drugs survey (ASSAD) monitoring survey. This study was approved by the Human Research Ethics Committee of Cancer Council Victoria (HREC 1013). Parental consent was required prior to their participation because this was a study of minors aged under 18 years of age. All guardians were sent home a consent form to sign which outlined the study purpose and the fact that responses were confidential. They were required to complete it and return it prior to their children commencing their survey. If guardian consent was not obtained, students were not asked to complete the survey.

The data are largely representative of the age levels sampled in the South Australian population. In 2011, a total sampling frame of 145 South Australian schools were approached; of these, 82 declined to participate, giving a final school participation rate of 43.5% (*n* = 63). A random sampling methodology was used to select schools from the Government, Catholic and Independent schools.^1^ Students were then randomly selected from within each identified school. Two samples were drawn to reflect junior students (up to Year 10) and senior students (Years 11–12). Several (16) primary schools were also included in the South Australian sample in order to obtain responses from Year 7 students (the majority of whom are 12 years old).

Participating schools provided the list of students currently enrolled for each of the year levels for which they were selected (junior or senior secondary), and random samples of 20 students (plus 6 replacement students) were identified. Survey researchers then attended the school to administer the pencil and paper questionnaire. Anonymity and confidentiality were emphasised during administration of the survey. A number of strategies were employed to enhance student perceptions of confidentiality, including use of external research staff, administering the survey under test conditions, placing teachers at the front or back of the room, training researchers only to look at questionnaires when asked a question by a student and providing blank envelopes into which students placed and sealed their questionnaires. They answered the survey anonymously and it was completed within one lesson. This was a monitoring survey and a power calculation for the hypotheses tested here was not therefore undertaken before data collection. Nonetheless, the sample size was such as to mitigate this as a limitation, a total of 2885 students aged between 12 and 17 completed the survey providing sufficient numbers for all between and within age group comparisons.

The national study was coordinated by Cancer Council Victoria, and approved by the Human Research Ethics Committee of Cancer Council Victoria (HREC 1013).

### Measures

#### Alcohol consumption

The primary dependent variable for this study was current drinking status. Students were asked “At the present time, do you consider yourself: A non-drinker; An occasional drinker; A light drinker; A party drinker; or A heavy drinker?” Responses were coded into *non-drinker* and *drinker* (all other categories) for most logistic regression analyses. Drinking status was collapsed because we wanted to test predictors of committed drinking behaviour versus no or episodic drinking. Students that indicated any consumption were asked to report the number of drinks that they had had in each of the last 7 days. Those that had had at least one drink in the last 7 days were classified as *recent drinkers*.

### Demographic and background variables

Students reported their age and gender and were asked, “During a normal week, how much money do you have available to spend on yourself (e.g. from pocket money, part-time job)?”. Response categories were ‘none’, ‘$1–$40’, ‘$41–$80’, ‘$81–$120’ and ‘$120 and over’. To assess self-rated performance at school, students were asked “At school work, do you consider yourself: ‘a lot above average?’, ‘above average’, ‘average’, ‘below average’, and ‘a lot below average’”. Students were asked to write the number of cigarettes they had each day for each of the last 7 days. Current smokers were classified as those that had smoked at least one cigarette in the past 7 days. Students reported their home postcodes, which were then matched with a corresponding Index of Relative Socio-economic Disadvantage quartile as a measure of neighbourhood socioeconomic status [28].

### Beliefs about drinking

Using the approach adapted from a previous study [29], students were asked to rate the importance of alcohol in increasing a person’s risk of getting cancer on a five- point scale (1 = not at all important, 2 = slightly important, 3 = moderately important, 4 = very important and 5 = extremely important). Responses were subsequently grouped into dichotomous categories combining “very” and “extremely important” versus all other responses for analyses.

Students were also asked to rate their agreement (1 = strongly disagree, 2 = disagree, 3 = agree, 4 = strongly agree) with the following statements: “My parents/guardian would not approve of me drinking” and “My friends would approve of me drinking”. Drinkers were also asked to rate agreement with the statement, “Being able to buy alcohol easily encourages me to drink a lot” using the same response format.

### Statistical analyses

Statistical analyses were undertaken using StataIC 13.1, the estimating tools of which account for the clustered, stratified survey design by utilising more robust estimates of standard error. Whether a school was a Government School, Catholic School or Independent School defined the strata. Chi-square tests were undertaken to determine univariate associations with ‘drinking’ i.e. ever (Yes/No) and ‘recent drinking’ (last 7 days) (Table 2). We undertook logistic regression models to determine predictors of these behaviours, stratified by age, to test the second hypothesis, specifically, that perceived parental disapproval for alcohol consumption will be protective in early years, and perception of peer approval of drinking would add risk in later schooling years (Tables 3, 4, 5). We then investigated recent drinking as the outcome variable among the sub-sample of drinkers (Table 6). Demographic variables were entered in both models (i.e., test of predictors of drinking status and test of predictors of drinking recently) at the first step. These included sex, the index of disadvantage, available spending money each week and self-reported ability at school. Smoking status was added at the second step. In the third step, awareness of the link between alcohol and cancer was added to test hypothesis 1, and finally friends’ approval and parental disapproval to test hypothesis 2. An additional variable was added into the drinkers status model: ‘being able to buy alcohol easily encourages me to drink a lot’, in order to determine whether availability was a significant additional predictor.

## Results

### Alcohol consumption among school students

Table 1 shows drinking status by age and gender. Reports of drinking (occasional through to heavy drinker) increased significantly with age (χ2 (df = 20, *N* = 2864) =715.78, *p* < .001) from 7.6% among 12 year olds to 66.3% among 17 year olds. Overall, 33.1% of students aged 12–17 years reported that they drank at least occasionally (95% CI = 31.0–35.2). There were more non-drinkers than drinkers among 12–15 year olds, but at 16 years of age the number of drinkers (59.5%) exceeded the number of non-drinkers. There was no significant difference by gender. Overall, 15.0% reported having consumed an alcoholic beverage in the past 7 days; this finding did not differ significantly by gender either (15.3% males and 14.8% females).

**Table 1:** Drinking status by age groups and gender

Drinking status	Males (%)	Females (%)	12–13 year olds (%)	14–15 year olds (%)	16–17 year olds (%)	Total (%)
	*n* = 1453	*n* = 1402	*n* = 968	*n* = 992	*n* = 894	*N* = 2855
Non-drinker	67.4	66.3	91.5	69.4	37.5	66.9
Occasional drinker	16.0	14.9	5.3	16.1	25.7	15.4
Light drinker	3.1	3.5	1.8	3.2	5.1	3.3
Party drinker	12.5	15.0	1.1	10.7	30.8	13.7
Heavy drinker	1.0	0.3	0.3	0.6	1.0	0.6
Total	100.0	100.0	100.0	100.0	100.0	100.0

### Perceptions of the link between alcohol and cancer

In total, 28.5% of students rated alcohol as “very important” or “important” in increasing a person’s risk of cancer. Males were less likely to rate alcohol as important or very important than females [23.6% vs. 33.5%, χ2 (df = 48, *N* = 2875) =34.4, *p* < .001]. Table 2 shows that although there was no significant difference in ratings of alcohol as a risk factor for cancer between 12 and 13 year olds who drank and those who did not, those aged 14–15 and 16–17 who drank were significantly less likely than those who did not drink to rate alcohol as “very important” or “important” in increasing a person’s risk of cancer.

**Table 2:** Cross-tabulations of various indicators by drinking status (at all drinkers and drank in the last 7 days) by age groups

	At all drinkers												Drank in the last 7 days among drinkers			
	12 & 13 year olds				14 & 15 year olds				16 & 17 year olds							
	Non-drinker % *n* = 886	Drinker % *n* = 82	*p*	φ_c_	Non-drinker % *n* = 688	Drinker % *n* = 304	*p*	φ_c_	Non-drinker % *n* = 335	Drinker % *n* = 559	*p*	φ_c_	Didn’t drink % *n* = 576	Drank % *n* = 379%	*p*	φ_c_
Smoked in last week	0.6	6.9	***	0.17	1.3	11.8	***	0.24	1.7	12.8	***	0.18	5.0	22.5	***	0.26
Index of disadvantage																
1st quintile	17.4	16.3			17.6	22.0			25.5	28.1			23.6	28.0		
2nd quintile	14.4	7.2			17.1	16.7			21.9	22.3			18.2	20.3		
3rd quintile	32.4	40.1			33.4	35.3			23.8	25.7			32.7	25.7		
4th quintile	19.1	16.0			12.8	10.7			14.1	12.8			11.5	13.6		
5th quintile	16.7	20.5	NS	NA	19.1	15.3	NS	NA	14.7	11.1	NS	NA	14.1	12.4	NS	NA
Available spending money per week																
None	18.3	12.2			13.6	5.8			10.4	4.2			6.3	4.2		
$1–$40	69.2	60.1			63.3	55.2			45.4	34.1			45.4	41.2		
$41–$80	4.7	11.8			11.7	17.5			18.2	20.8			19.4	18.3		
$81–$120	2.8	9.7			3.8	8.5			12.3	16.7			13.5	13.1		
$121 and over	5.1	6.3	*	0.12	7.7	13.0	***	0.18	13.7	24.2	***	0.20	15.4	23.3	*	0.09
Self-reported ability at school																
A lot above average	4.4	7.9			6.3	3.7			9.6	3.3			3.9	3.6		
Above Average	35.2	19.0			37.3	30.6			38.9	32.6			31.5	29.8		
Average	52.1	53.3			52.4	56.1			46.5	55.1			56.6	53.5		
Below average	8.1	17.9			3.6	7.8			4.9	7.6			7.0	10.8		
A lot below average	0.2	1.9	**	0.13	0.3	1.8	**	0.14	0.2	1.4	***	0.17	1.0	2.4	NS	NA
Parental disapproval (Strongly/ disagreed)	75.3	36.9	***	0.20	75.9	58.7	***	0.18	70.5	45.3	***	0.24	51.3	45.7	NS	NA
Friends approval (strongly/agreed)	22.4	65.6	***	0.25	46.8	85.0	***	0.37	61.7	91.6	***	0.38	87.5	88.3	NS	NA
Alcohol and cancer (very/important)	28.3	33.7	NS	NA	31.4	23.8	*	0.08	34.2	23.4	***	0.11	26.3	21.7	NS	NA
Being able to buy alcohol easily encourages me to drink a lot													17.8	27.7	***	0.12
Age																
12–13													11.8	6.1		
14–15													31.3	33.4		
16–17													56.9	60.5	NS	NA

*** *p* ≤ 0.001, **p ≤ 0.01, * *p* < 0.05 chi square, NS = Not statistically significant at *p* < 0.05, NA = Not applicable

### Predictors of alcohol consumption

A number of the demographic variables and the measures of reported endorsement of drinking by others were significantly related to drinking status at the univariate level (Table 2). There was a clear association between smoking and drinking across all ages. There was no clear association between drinking and the index of disadvantage. Those with more available spending money per week were more likely to drink at all ages as were those with average or below average self-reported ability at school. Parental disapproval was protective from consumption whereas friends’ approval was predictive of consumption (at all). Those that thought there was an important link between alcohol and cancer were less likely to drink when aged 14–17 years. Those that agreed that “being able to buy alcohol easily encourages me to drink a lot” were more likely to drink. Overall, 4 models were tested for each of the three age groups (12–13 year olds: Table 3, 14–15 year olds: Table 4 and 16–17 year olds: Table 5).

**Table 3:** Logistic regression analysis: significant predictors of drinking alcohol for 12–13 year olds (Level 1 as reference category)

	Model 1 OR *n* = 950	95% CI	Model 2 OR *n* = 945	95% CI	Model 3 OR *n* = 945	95% CI	Model 4 OR *n* = 525	95% CI
Sex (ref:male)	0.68	0.35–1.33	0.64	0.33–1.25	0.62	0.31–1.22	0.49	0.21–1.15
Index of disadvantage (ref: most disadvantage)								
2nd quintile	0.54	0.11–2.73	0.60	0.12–3.06	0.60	0.12–2.99	0.07	0.00–1.22
3rd quintile	1.25	0.59–2.66	1.46	0.71–3.02	1.46	0.70–3.04	1.10	0.46–2.62
4th quintile	0.94	0.40–2.20	0.93	0.39–2.21	0.95	0.40–2.25	1.50	0.31–7.40
5th quintile	1.35	0.70–2.61	1.61	0.85–3.02	1.62	0.87–3.01	0.63	0.19–2.13
Available spending money per week (ref: none)								
$1–$40	1.28	0.69–2.37	1.34	0.71–2.55	1.36	0.72–2.58	3.18	0.70–14.47
$41–$80	3.52*	1.13–10.97	3.82*	1.23–11.83	3.76*	1.21–11.72	9.10*	1.72–48.04
$81–$120	4.12*	1.11–15.26	3.36	0.75–15.12	3.30	0.78–13.96	13.14**	2.36–73.31
$121 and over	1.63	0.31–8.51	1.72	0.31–9.59	1.77	0.33–9.60	3.73	0.26–53.65
Self-reported ability at school (ref: a lot above average)								
Above average	0.36	0.09–1.38	0.32	0.08–1.18	0.31	0.09–1.16	0.16*	0.03–0.74
Average	0.61	0.17–2.20	0.57	0.17–1.97	0.58	0.17–2.01	0.45	0.10–2.09
Below average	1.17	0.26–5.31	0.91	0.22–3.76	0.92	0.22–3.92	0.84	0.17–4.27
A lot below average	4.15	0.29–59.97	1.34	0.16–11.52	1.33	0.16–11.44	0.66	0.04–9.69
Smoked in last week (ref: no)			10.33**	2.87–37.16	10.64**	3.02–37.46	15.04*	1.97–114.79
Alcohol and cancer (ref: very/important)					0.77	0.41–1.44	0.42*	0.18–0.97
Friends approval (ref: no)							6.02**	1.91–18.93
Parental disapproval (ref: no)							0.25**	0.11–0.55

***p ≤ 0.001, **p ≤ 0.01, *p < 0.05

**Table 4:** Logistic regression analysis: significant predictors of drinking alcohol for 14–15 year olds (Level 1 as reference category)

	Model 1 OR *n* = 961	95% CI	Model 2 OR *n* = 956	95% CI	Model 3 OR *n* = 956	95% CI	Model 4 OR *n* = 598	95% CI
Sex (ref:male)	1.04	0.74–1.48	1.06	0.75–1.51	1.10	0.78–1.57	1.42	0.90–2.23
Index of disadvantage (ref: most disadvantage)								
2nd quintile	0.76	0.42–1.39	0.80	0.44–1.44	0.81	0.44–1.47	0.88	0.41–1.89
3rd quintile	0.80	0.48–1.33	0.79	0.47–1.33	0.80	0.47–1.37	0.70	0.36–1.35
4th quintile	0.70	0.42–1.14	0.66	0.42–1.04	0.67	0.42–1.06	0.89	0.53–1.49
5th quintile	0.66	0.40–1.08	0.66	0.41–1.06	0.66	0.41–1.07	0.49*	0.25–0.97
Available spending money per week (ref: none)								
$1–$40	2.13*	1.19–3.82	2.05*	1.15–3.63	2.01*	1.13–3.57	1.37	0.68–2.76
$41–$80	3.71**	1.86–7.43	3.65**	1.80–7.42	3.64**	1.77–7.53	2.05	0.75–5.63
$81–$120	5.76***	3.28–10.12	5.52***	3.27–9.32	5.22***	3.11–8.77	2.18	0.91–5.24
$121 and over	4.31***	2.32–7.99	4.47***	2.40–8.32	4.43***	2.41–8.15	1.81	0.75–4.36
Self-reported ability at school (ref: a lot above average)								
Above average	1.79	0.80–3.99	1.72	0.78–3.79	1.74	0.79–3.84	2.38	0.85–6.64
Average	2.27*	1.13–4.54	1.98*	1.01–3.86	1.92	0.98–3.74	1.92	0.70–5.24
Below average	5.82***	2.41–14.02	4.47**	1.80–11.10	4.29**	1.73–10.66	4.33*	1.20–15.67
A lot below average	12.45*	1.76–87.91	6.09	0.75–49.31	5.94	0.69–51.48	0.42	0.05–3.56
Smoked in last week (ref: no)			9.91***	3.96–24.81	9.96***	3.96–25.08	10.07**	2.49–40.68
Alcohol and cancer (ref: very/important)					1.43**	1.05–1.94	1.65*	0.99–2.74
Friends approval (ref: no)							6.25***	4.22–9.27
Parental disapproval (ref: no)							0.56**	0.37–0.84

***p ≤ 0.001, **p ≤ 0.01, *p < 0.05

**Table 5:** Logistic regression analysis: significant predictors of drinking alcohol for 16–17 year olds (Level 1 as reference category)

	Model 1 OR *n* = 874	95% CI	Model 2 OR *n* = 871	95% CI	Model 3 OR *n* = 871	95% CI	Model 4 OR *n* = 641	95% CI
Sex (ref:male)	1.16	0.84–1.61	1.15	0.82–1.62	1.23	0.87–1.74	1.54*	1.01–2.32
Index of disadvantage (ref: most disadvantage)								
2nd quintile	0.98	0.63–1.53	1.03	0.66–1.62	1.04	0.67–1.61	1.32	0.78–2.22
3rd quintile	1.00	0.66–1.52	0.95	0.63–1.43	0.93	0.63–1.38	0.90	0.55–1.48
4th quintile	0.87	0.55–1.39	0.90	0.56–1.46	0.92	0.57–1.47	0.71	0.42–1.19
5th quintile	0.76	0.45–1.26	0.79	0.48–1.30	0.76	0.47–1.24	0.50**	0.31–0.78
Available spending money per week (ref: none)								
$1–$40	1.98**	1.20–3.26	2.08**	1.23–3.48	2.12**	1.25–3.61	2.18*	1.21–3.91
$41–$80	3.04***	1.79–5.19	3.33***	1.99–5.57	3.38***	2.00–5.74	3.03**	1.57–5.82
$81–$120	3.47**	1.76–6.83	3.38**	1.68–6.83	3.41***	1.68–6.92	2.57*	1.21–5.43
$121 and over	4.67***	2.60–8.39	4.99***	2.77–8.96	4.97***	2.73–9.03	4.78***	2.32–9.84
Self-reported ability at school (ref: a lot above average)								
Above average	2.42*	1.18–4.97	2.35*	1.18–4.68	2.29*	1.13–4.63	1.81	0.94–3.48
Average	3.52**	1.75–7.09	3.27**	1.65–6.48	3.08**	1.53–6.16	2.73**	1.37–5.45
Below average	4.40**	1.91–10.12	3.74**	1.67–8.36	3.30**	1.42–7.69	3.63*	1.13–11.56
A lot below average	18.15*	1.48–221.91	9.25	0.62–137.07	9.73	0.63–149.41	–	–
Smoked in last week (ref: no)			8.97***	4.52–17.82	8.94***	4.52–17.67	7.88***	3.09–20.10
Alcohol and cancer (ref: very/important)					1.68**	1.24–2.29	1.82**	1.23–2.69
Friends approval (ref: no)							6.94***	4.19–11.49
Parental disapproval (ref: no)							0.41***	0.29–0.57

***p ≤ 0.001, **p ≤ 0.01, *p < 0.05

Among 12–13 year olds, model 1 showed that school children at this age are 3.5 times more likely to drink if they have between $41 and $80 spending money per week, and 4 times more likely to drink if they have between $81 and $120. Gender, socio-economic disadvantage and self-reported schooling ability were not significant influences on drinking. In model 2, smokers were 10 times more likely to drink. Model 3 showed that the addition of awareness of the link between alcohol and cancer did not explain additional variance. Model 4 included friends’ approval of drinking and parental disapproval of drinking. The addition of these variables increased the odds ratios for smokers to 15 times more likely to drink and available spending money to 9 times more likely, for those with between $41 and $80 a week, and 13 times for those with $81–$120 per week. Interestingly, the inclusion of these variables resulted in a significant incremental contribution to predicted variance by cancer-alcohol knowledge; those who did *not* link alcohol and cancer were *less likely to drink*, a direction contrary to that hypothesised. Consistent with the hypothesis, those who reported that their friends approved of their drinking were 6 times more likely to drink and those who reported that their parents did not approve of drinking were much less likely to drink.

The picture among 14–15 year olds was very similar, although the influence of schooling ability was significant in this age group. Model 1, Table 4 shows that amount of available spending money increased the odds of drinking by at least 2 times up to nearly 6 times for those with between $81 and $120 per week. Those reporting average schooling ability were twice as likely as those that were a lot above average to drink; those that were below average were nearly 6 times and those that reported they were a lot below average were 12 times more likely to drink. Model 2 added smoking status, which did not change the other odds ratios much, but did confirm that smokers were nearly 10 times more likely to drink. Model 3 added awareness of the link between alcohol and cancer, which was significant. The other odds ratios did not change much, but those that did not see alcohol as a very or important risk factor for cancer were about 1.5 times more likely to drink alcohol as those that did see it as a risk, confirming the hypothesis within this age group. Model 4 included friends’ approval of drinking and parental disapproval of drinking. The inclusion of these variables reduced the importance of available spending money as a predictor to non-significance, but all other odds ratios remained similar. Consistent with the hypothesis, those who reported that their friends approved of their drinking were 6 times more likely to drink and those who reported that their parents did not support their drinking were less likely to drink.

A similar result was found among 16 and 17 year olds (Table 5). Model 1 showed that available spending money increased the odds of drinking by at least two times, and up to 4.7 times for those getting over $121 per week. Those reporting above average schooling ability were twice as likely as those that were a lot above average to drink; those that were average were 3.5 times, those below average were 4.4 times and those that reported they were a lot below average were 18 times more likely to drink. Model 2 added smoking status, which did not substantially change the other odds ratios, but removed the effect of being a lot below average ability and indicated that smokers were nearly 9 times more likely to drink than those that did not smoke. Model 3 added awareness of the link between alcohol and cancer, which was significant. The other odds ratios changed very little, but those respondents that did not see alcohol as a very or important risk factor for cancer were 1.7 times more likely to drink alcohol compared to those that did, consistent with the hypothesis. Model 4 included friends’ approval of drinking and parental disapproval of drinking. Contrary to the hypothesis, the inclusion of these two variables did not substantially change the other odds ratios but allowed for the detection of gender as a possible predictor in the model. Those that were least disadvantaged were less likely to drink, as were those who reported that their parents did not support their drinking (contrary to that hypothesised for older age groups), while those who reported that their friends approved of their drinking were nearly 7 times more likely to drink.

Predictors for drinking alcohol in the past week (i.e. recent drinking) were also examined (Table 6). Model 1 shows that those aged 14–15 years old were twice as likely as 12–13 year olds to drink in the previous week. Those in the third quintile of disadvantage were less likely to drink regularly than the most disadvantaged group. Those with access to available spending money of $121 and over were also 2.3 times more likely to drink recently than those with no available spending money. Model 2 added smoking status, which was again a strong predictor (nearly 6 times more likely to drink regularly). Contrary to hypothesis one and two, the addition of awareness of the link between alcohol and cancer did not add to the model, nor did friends’ approval of drinking and parental disapproval. Finally, those that agreed that ‘being able to buy alcohol easily encourages me to drink’ were 1.7 times more likely to be recent drinkers.

**Table 6:** Logistic regression analysis: significant predictors of drinking alcohol in the last 7 days among drinkers (Level 1 as reference category)

	Model 1 OR *n* = 931	95% CI	Model 2 OR *n* = 926	95% CI	Model 3 OR *n* = 926	95% CI	Model 4 OR *n* = 690	95% CI	Model 5 OR *n* = 619	95% CI
Age (ref 13, 14 year olds)										
14–15 year olds	2.09*	1.01–4.36	1.90	0.95–3.80	1.88	0.94–3.76	1.38	0.55–3.44	1.44	0.48–4.30
16–17 year olds	1.91	0.99–3.70	1.65	0.88–3.12	1.64	0.87–3.09	1.19	0.51–2.78	1.25	0.43–3.63
Sex (ref: male)	1.01	0.79–1.30	1.08	0.82–1.41	1.09	0.83–1.42	0.96	0.71–1.30	0.94	0.68–1.30
Index of disadvantage (ref: most disadvantage)										
2nd quintile	0.97	0.64–1.47	1.00	0.65–1.52	1.00	0.65–1.53	1.03	0.61–1.71	0.91	0.52–1.59
3rd quintile	0.67*	0.49–0.93	0.62*	0.44–0.87	0.62**	0.44–0.88	0.76	0.51–1.12	0.76	0.52–1.10
4th quintile	1.09	0.66–1.79	1.13	0.69–1.85	1.13	0.69–1.85	1.19	0.70–2.02	1.07	0.59–1.92
5th quintile	0.82	0.49–1.38	0.87	0.51–1.48	0.87	0.51–1.48	1.04	0.53–2.06	0.91	0.45–1.86
Available spending money per week (ref: none)										
$1–$40	1.40	0.76–2.57	1.58	0.80–3.13	1.58	0.80–3.12	1.23	0.56–2.70	1.05	0.46–2.38
$41–$80	1.46	0.70–3.04	1.71	0.76–3.89	1.71	0.75–3.89	1.39	0.54–3.54	1.29	0.51–3.30
$81–$120	1.45	0.73–2.86	1.48	0.70–3.10	1.48	0.70–3.10	1.18	0.49–2.84	0.94	0.41–2.15
$121 and over	2.35**	1.24–4.42	2.79**	1.38–5.60	2.78**	1.38–5.58	2.56*	1.10–5.95	1.89	0.82–4.33
Self-reported ability at school (ref: a lot above average)										
Above average	0.89	0.51–1.54	0.86	0.48–1.54	0.86	0.48–1.54	0.86	0.40–1.84	0.78	0.35–1.75
Average	0.91	0.47–1.76	0.79	0.39–1.60	0.79	0.39–1.59	0.76	0.32–1.82	0.58	0.23–1.43
Below average	1.64	0.77–3.50	1.23	0.55–2.76	1.22	0.55–2.72	0.97	0.35–2.72	0.81	0.29–2.30
A lot below average	2.37	0.71–7.90	1.13	0.39–3.31	1.14	0.39–3.32	0.82	0.18–3.75	0.62	0.12–3.34
Smoked in last week (ref: no)			5.89***	3.36–10.34	5.86***	3.33–10.31	5.17***	2.72–9.84	4.00***	2.11–7.56
Alcohol and cancer (ref: very/important)					1.09	0.77–1.55	1.11	0.75–1.65	1.13	0.75–1.70
Friends approval (ref: no)							1.08	0.62–1.86	1.04	0.60–1.79
Parental disapproval (ref: no)							0.74	0.54–1.01	0.74	0.54–1.02
Being able to buy alcohol easily encourages me to drink a lot (ref: no)									1.70*	1.09–2.65

***p ≤ 0.001, **p ≤ 0.01, *p < 0.05

## Discussion

The first aim of this study was to confirm the prevalence of alcohol consumption. A pattern of increasing consumption with age, reported in previous literature, was observed in our data. By 16 years of age the number of drinkers exceeded the number of non-drinkers.

The second hypothesis of the study was that the majority of students would not be aware that alcohol causes cancer but this was only partially supported with results varying between age groups and outcomes.

Overall, the results revealed that, as hypothesed, awareness of the link was low, with only one in four or 28.5% of students being aware. These figures were lower than those obtained in a recent survey of Australian adults (36.6%) [19], and of the finding in the UK that 37% of young people aged 15 to 24 years were aware of the link [18]. It is important to note that since this study was undertaken, awareness may have increased as evidence of a clear link between alcohol and cancer has improved and dissemination increased. It is beyond the scope of this study to investigate paid and unpaid media on this topic, but it warrants further research and a follow-up study should be undertaken.

Consistent with previous research involving adults [19], results indicated that awareness of the cancer link discriminated ‘no consumption ever’ from ‘any consumption’, although, paradoxically, we found no relationship between awareness and recent consumption. It is possible that such a relationship (even though it may be small) might exist among recent drinkers, but that low levels of recent consumption in the current sample, notwithstanding the large sample size, obscured this relationship. It was also interesting to note that while awareness of the link between alcohol and cancer was protective against drinking in the 14–17 year olds, the inverse relationship was found for 12–13 year olds. This may be a statistical anomaly or may indicate that awareness of the link between alcohol and cancer becomes more important with age.

The second hypothesis of this study was that perceived parental disapproval for alcohol consumption will be protective in early years, and perception of peer approval of drinking will predict consumption in later school years. The results indicated that students’ perceptions of parental disapproval was predictive of drinking at all mid-teen ages (i.e. where a student reported that a parent did not disapprove, a student was more likely to consume alcohol). This contrasts with predictions based on Social Development Theory that parental attitudes would have reduced importance with increasing adolescent age. Moreover, also in contrast with theory, perceptions of peer attitudes to alcohol was a significant predictor regardless of age.

As expected, and consistent with previous studies [26], smoking was strongly related to alcohol consumption across ages. It would be interesting to explore whether the awareness of cancer-alcohol risk differs for smokers and non-smokers because it is possible that smokers may weigh cancer risk less heavily in their decision-making. Also consistent with the literature, available spending money per week [24] and self-reported average or below-average school achievement were predictive of drinking [25]. Interestingly, however, when non-drinkers were removed from the model, the only significant predictors of drinking within the previous week were smoking status and the perception that ‘being able to buy alcohol easily encourages me to drink a lot’. This result presents some difficulty for interpretation given that ease might relate to any one or more of financial ease, physical accessibility, or parental attitude. Further research should examine the drivers of self-reported ease of access.

The strengths of the current study include the large sample size and the fact that data were weighted to reflect the South Australian school student population, increasing confidence in the generalisability of the findings. There are, however, some limitations. The survey was cross-sectional so causation cannot be determined. In addition, the study relies on self-report of drinking identity rather than actual behaviour; this can be subject to bias, although anonymity was assured to reduce this potential bias. The study looked at drinking frequency rather than volume of consumption, and amounts consumed should be investigated in future studies. Also, while the questionnaire had face validity its questions have not undergone rigorous reliability and validity testing. The survey was also limited by the small number of predictors able to be included because of cost and time restrictions; we did not assess the role that other factors might play including media, advertising and role modelling.

## Conclusions

The present study demonstrates that consumption of alcohol by adolescents increases with age cross-sectionally. Moreover, consumption can be predicted by adolescent report of parental attitudes towards drinking, and also by peer attitudes. In light of the findings that early initiation of drinking predicts poorer health in later life [8], the study highlights the importance of parental attitudes to student drinking. It is also interesting to note that those aged 14–17 years with an awareness of the link between alcohol and cancer were less likely to drink, indicating that an education campaign and messaging about the link might impact young people. It would therefore be potentially beneficial for schools to include greater information concerning the longer-term health consequences of drinking in health advice provided during the school years. The study’s results also indicate that greater consideration should be given to the factors that serve to regulate accessibility to alcohol among those underage.

## Abbreviation

Ref: Reference category
SES: Socioeconomic status

## Footnotes

1 The Australian schooling system consists of primary school (generally for ages 5 to 12 years) and then secondary school (generally age 12 to 17 years). Most Catholic schools are run by their local parish, local diocese and the Catholic education department. The majority of other independent schools have a formal religious affiliation (e.g. Protestant, Jewish, Islamic), while some are non-denominational. Some pursue particular educational philosophies (e.g., Montessori or Steiner educational philosophies). Although all types of school receive federal government support, many independent schools charge fees for attendance.
